# The EQo-Mental project: A protocol for a mixed-methods study on occupational balance and mental health in parents of children with developmental delays

**DOI:** 10.1371/journal.pone.0337542

**Published:** 2025-12-01

**Authors:** Desirée Valera-Gran, Miriam Hurtado-Pomares, Iris Juárez-Leal, Rocío Muñoz-Sánchez, Irene Campos-Sánchez, Paula Noce, Jessica Piñero, Eva-María Navarrete-Muñoz

**Affiliations:** 1 Department of Surgery and Pathology, Miguel Hernández University, San Juan de Alicante, Spain; 2 Occupational Therapy Research Group (InTeO, Investigación en Terapia Ocupacional), Miguel Hernández University, San Juan de Alicante, Spain; 3 Institute for Health and Biomedical Research of Alicante (ISABIAL, Instituto de Investigación Sanitaria y Biomédica de Alicante), Alicante, Spain; 4 Education Faculty, International University of La Rioja, Logroño, Spain; 5 Neurodevelopment Research Center, Fundación Salud Infantil, Elche, Spain; 6 Joint Research Unit UMH-Fisabio (STATSALUT), Elche, Spain; Murcia University, Spain, SPAIN

## Abstract

**Background:**

Parents of children with developmental delays (DD) often face significant challenges that affect their mental health and occupational balance. While early intervention services traditionally focus on child development, the occupational needs and well-being of parents remain underexplored. The EQo-Mental project aims to examine the association between parental mental health, occupational balance, and meaningful activity engagement, and to co-develop family-centred strategies that promote well-being in early intervention contexts.

**Methods:**

This sequential mixed-methods study includes two phases. The quantitative phase will involve approximately 700 parents of children aged 0–6 years attending early intervention centres in Alicante, Spain. This phase comprises two components: (1) the psychometric validation of the Spanish versions of two occupational measures—the Occupational Balance Questionnaire (OBQ-E) and the Engagement in Meaningful Activities Survey (EMAS)—and (2) a cross-sectional analysis examining associations between occupational and mental health outcomes. Participants will complete a sociodemographic questionnaire along with validated self-administered instruments assessing occupational balance, meaningful activity engagement, stress, anxiety, depression, and psychological well-being. In the qualitative phase, participatory sessions and focus groups will be conducted with a subsample of parents and key stakeholders to explore perceived occupational and mental health needs and to co-design actionable strategies for improving occupational balance and family well-being. Participant recruitment began in November 2023 and is ongoing; data collection is expected to be completed by October 2025.

**Analyses:**

Psychometric analyses will first be conducted to evaluate the validity and reliability of the OBQ-E and EMAS. Next, descriptive analyses and multiple regression models adjusted for potential confounders will be used to explore associations between occupational and mental health variables. Phase 2 consists of a participatory-action research process, including discussion groups and a multi-stakeholder focus group. Qualitative data will be analysed using reflexive thematic analysis.

**Outcomes:**

Findings from EQo-Mental will inform the design of evidence-based, family-centred strategies that support occupational balance, parental well-being, and engagement in meaningful activities. By addressing the occupational needs of parents, the project seeks to foster more resilient families and strengthen early intervention services through an inclusive, occupation-focused approach.

## Introduction

Children with developmental delays (DD) constitute a broad and heterogenous group affected by a wide range of adverse health conditions. These children may exhibit delays, regressions, or losses in two or more developmental domains, including gross and fine motor skills, speech and language, cognition, social and personal skills, and activities of daily living [1,2]. They frequently encounter sensory impairments, such as hearing and vision loss, along with conditions such as epilepsy, cerebral palsy, attention deficit hyperactivity disorder (ADHD), autism spectrum disorder (ASD), intellectual disability, or various learning disorders [2,3].

The early years of life represent a critical window for achieving key developmental milestones [4], making timely diagnosis and intervention essential. When DD is identified early and followed by an appropriate intervention, children are more likely to enhance their developmental potential, functional performance, and overall quality of life, while fostering their opportunities for social participation [5].Although caring for children with DD can bring positive familial outcomes [6] such as enhanced family cohesion, personal development, and increased joy [7], research has also consistently documented the chronic stressors that parents often face. These stressors may include high caregiving demands [8–10], social stigma [10,11], sleep deprivation [10,11], limited social contact [10–12], disruptions to work schedule [10] and family responsibilities [10,11], and financial burden of treatment [10,11]. Each of these factors can contribute to deteriorating mental health in parents [7,9–13].

Literature focused on the challenges experienced by parents of children with DD has highlighted that the burden of caregiving frequently leads to increased levels of stress [10–12,14,15], anxiety [7,10,11,14,16] and depression [7,10–12]. Several studies have identified major sources of parental distress, particularly concerns about their child’s health and developmental prognosis [10,17], often embedded in daily routines involving recurrent therapies, assessments, and specialised educational support [10,18]. This scenario usually becomes worse because parents tend to prioritise caregiving responsibilities over personal needs, eventually leading to an increased opportunity for occupational imbalance marked by the loss of meaningful activities [19–22], reduced social interaction and support, and inadequate self-care further exacerbating their psychological distress [10,23]. Depression, in particular, frequently co-occurs with stress and anxiety in this population [7,10]. A meta-analysis by Scherer et al. (2019), which included 3303 parents of children with DD and 9519 parents of children without DD across 11 countries, found that approximately one-third (31%) of the parents with children with DD reported elevated levels of depressive symptoms [7].These findings underscore the magnitude of the caregiving burden and highlight the need to better understand the risks factors associated with the parenting role in such context and their impact on occupational performance, understood as the ability to engage in meaningful and necessary daily activities that support health, well-being, and social participation [24].

A significant shift in family roles and increased caregiving demands may constitute two of the most challenging and interconnected experiences for parents of children with DD. These circumstances often disrupt established family dynamics and parental roles, intensifying caregiving responsibilities and requiring parents to juggle additional familial and social commitments [10,25]. These increased demands can reduce the time available for meaningful activities, which are defined as everyday activities undertaken individually that hold intrinsic meaning and purpose, encompassing necessary, desired, and expected actions [19,26]. A reduction or withdrawal of such activities may negatively affect occupational balance, understood as the perception of having a satisfactory and varied engagement in meaningful activities throughout daily life [27,28]. Notably, these burdens are often disproportionately borne by women, who typically assume the primary caregiving role within families. This unequal distribution of care has been linked to heightened psychological distress, including anxiety and depression among mothers of children with DD [29–31]. Additionally, it may further aggravate occupational imbalance by limiting time for rest, social participation, and self-care [32].

Although several studies have documented perceived disruptions in occupational balance as a detrimental consequence of informal caregiving [21,33–35], there remains a lack of empirical research supporting the hypothesis that occupational balance might impact on life satisfaction, well-being and quality of life of parents of children with DD [19,22,34,36].

To our knowledge, no scientific studies have specifically examined the interplay between psychological distress, well-being, and occupational performance factors—such as engagement in meaningful activities and occupational balance—and their impact on the health of parents of children with DD. This gap in evidence is compounded by the lack of validated instruments to assess these constructs in this population, which limits the ability to monitor caregiver burden and guide tailored interventions. Consequently, the development of strategies that address parents’ occupational needs and promote occupational balance remains limited. In response, identifying and co-designing supportive actions through participatory research becomes essential to generate meaningful interventions that strengthen parents’ occupational balance and well-being, ultimately contributing to healthier developmental environments for their children.

Our project represents the first attempt in Spain to comprehensively explore these associations. The findings may inform the development of family-centred policies and practices in early intervention centres, supporting not only children but also addressing the occupational needs of their parents. Enhancing parental satisfaction with daily activities and promoting occupational balance could contribute to improved emotional well-being.

Therefore, the main aim of this study is to examine mental health, engagement in meaningful activities, and occupational balance among parents of children with DD. Specifically, we aim to:

1. Evaluate the psychometric properties of the Spanish versions of the Occupational Balance Questionnaire (OBQ-E) [37] and the Engagement in Meaningful Activities Survey (EMAS) [38] in parents of children with DD.
2. Describe perceived symptoms of stress, anxiety and depression, as well as psychological well-being in this population.
3. Assess levels of engagement in meaningful activities and occupational balance.
4. Identify the most frequently identified meaningful activities by parents of children with DD.
5. Explore factors sociodemographic, lifestyle, and contextual associated with stress, anxiety, depression, psychological well-being, engagement in meaningful activities, and occupational balance.
6. Examine the association between engagement in meaningful activities and occupational balance and mental health outcomes (anxiety, depression, stress, and psychological well-being).
7. Identify the needs and potential actions, through a participatory process, to improve involvement in meaningful activities and occupational balance among these parents.

## Materials and methods

### Study design, participants, and setting

The EQo-Mental (*Equilibrio ocupacional y salud Mental*, Occupational balance and Mental health) project is a mixed-methods, combining quantitative and qualitative approaches to address the study objectives, and structured into two distinct phases. The first phase involves the collection and analysis of quantitative data, while the second phase focuses on a participatory process. Participants will be parents of children with DD, aged 0–6 years, who are currently attending public or private early intervention centres in the province of Alicante, Spain. Public centres were selected with the Department of Equality and Inclusive Policies of the Generalitat Valenciana (https://serviciossociales.gva.es/es/web/discapacidad/centres-programes-atencio-primerenca), while private centres were located though an active search conducted by the research team. To participate, parents must be competent Spanish speakers. Additional information about the project is available at https://inteo.umh.es/eqomental/.

### Ethical approval, considerations and dissemination

The EQo-Mental project aims to generate both quantitative evidence (via questionnaires) and qualitative insights (through participatory-action research). Prior to data collection, all participants will be fully informed about the study objectives and procedures, and written informed consent will be obtained from each participant.

The processing and protection of personal data will comply with the Spanish Organic Law 3/2018 of December 5, on the Protection of Personal Data and Guarantee of Digital Rights. The study protocol received favourable ethical approval from the Committee for Ethics and Research Integrity of the Miguel Hernández University of Elche (Ref. DPC.MHP.01.22), and all research procedures will adhere to the principles outlined in the Declaration of Helsinki.

### Sample size calculation

To evaluate the psychometric properties of the OBQ-E and EMAS, a total of 150 parents of children with DD will be recruited. To assess test-retest reliability, data will be collected at two different time points from half of the participants (n = 75). For concurrent validity, we applied the formula for detecting significant correlation coefficients: n = (Zα + Zβ)² × δ²/ d², using the Fisher Z transformation of correlation coefficients, where δ² = 1 for the Z-scale. Assuming a significance level of α = 0.05, power of 1–β = 0.80, and aiming to detect a correlation of at least 0.3, a minimum of 110 participants is required. This sample will allow sufficient power to detect moderate associations between the scales and related constructs.

To address the remaining objectives of quantitative study, a larger sample size is required. However, no comparable studies involving parents of children with DD were identified to inform an exact sample size calculation. Based on previous research on occupational balance in parents [39], and assuming a standard deviation of 8 points, a power of 80%, and a 95% confidence level, a sample of 246 parents would be required to describe occupational balance accurately.

Regarding mental health outcomes, the sample size was estimated based on the prevalence of 31% of anxiety and depression symptoms in parents of children with some DD, as reported by Scherer et al. (2019). Assuming a significance level of 5%, a power of 80% and a two-sided test, a sample of 329 participants would be optimal. However, given that multiple regression models will be conducted, a larger sample is essential. A common recommendation is to include at least 10–20 participants per predictor variable to ensure model stability, avoid overfitting, and improve generalisability [40]. Therefore, the study aims to collect data from approximately 700 participants across 20 early intervention centres (representing 30% of the children served in the province of Alicante). This sample size is expected to provide sufficient statistical power (80%) to detect β coefficients greater than 1.5 in complex multiple modelling and potential subgroup analyses.

### Enrolment and procedure

Early intervention centres will be invited to participate in the study via email or telephone contact. An informational session will subsequently be held at each centre to present the study aims and procedures to the centre’s management team. Recruitment of families will be facilitated through the child’s clinical care team and supported by informational materials (e.g., posters and brochures) placed in common areas.

Parents who express interest will be provided with detailed information about the study and asked to provide written informed consent. Once enrolled, participants will be scheduled for the quantitative data collection phase.

For the qualitative phase, centres will be selected using purposive sampling, prioritising those with higher participant enrolment in the quantitative phase to ensure sufficient representation and feasibility for group discussions. In each of these centres, a participatory group of approximately 4–10 parents will be assembled to take part in structured group sessions designed to explore needs, experiences, and possible actions to improve occupational balance and well-being.

### Data collection procedures for the quantitative phase

The quantitative phase of the EQo-Mental study includes two components: (1) the psychometric validation of the OBQ-E and EMAS, and (2) a cross-sectional study exploring associations between occupational and mental health variables. Both components share the same recruitment and data collection strategy, carried out in early intervention centres across the province of Alicante.

To validate the OBQ-E and EMAS, a minimum of 150 participants will be recruited, as recommended by the COSMIN group (COnsensus-based Standards for the selection of health Measurement INstruments) [41]. A subsample of approximately 75 participants will be invited to complete the OBQ-E and EMAS a second time after a 2–4-week interval to assess test–retest reliability. Participants for the retest will be selected based on availability and willingness to complete the follow-up assessment.

For the broader cross-sectional study, approximately 700 parents will be recruited. Parents will be invited to participate during their child’s scheduled visit to the centre. The assessment session will last approximately 45 minutes. During this session, an assessor will administer an ad hoc questionnaire and guide participants in completing the self-administered standardised assessments. Alternatively, parents may complete the questionnaires at home and return them via the clinical team. Any questions will be addressed by the assessor, either in person or by phone.

All participants will complete a sociodemographic and lifestyle questionnaire, along with validated instruments assessing occupational balance, meaningful activity engagement, stress, anxiety, depression, and psychological well-being. The same dataset will be used for both the psychometric and cross-sectional analyses, depending on the specific objectives and sample size requirements.

### Procedure for participatory-action research process (qualitative data)

This phase will use a participatory-action research approach. In the selected centres, groups of parents will be formed and provided with a safe space to share experiences, reflect collectively, and identify both needs and areas for improvement.

These sessions are intended to empower participants to contribute ideas and co-develop proposals for change. The insights gathered will be summarised in a final report and shared with the management teams of each centre. The proposed actions will aim to enhance engagement in meaningful activities, promote occupational balance, and ultimately support the emotional health and well-being of families. In this phase, parents will act as active contributors and co-researchers, playing a central role in designing interventions from a family- and community-centred perspective.

### Study measures

Sociodemographic, health, and lifestyle data will be collected using a combination of ad hoc questionnaires and standardised instruments. The questionnaires were adapted from those used in the InProS study (https://inteo.umh.es/inpros/) and complemented with validated measures. A summary of the variables and instruments used in the EQo-Mental project is presented in Table 1.

**Table 1:** Summary of the data collection for the EQo-Mental project.

Outcome measures	Participants		Measurement method
	Parents	Child	
**Occupational participation**			
Occupational balance	x		OBQ-E
Meaningful activities	x		EMAS
**Mental health**			
Stress	x		Parental Stress scale
Anxiety	x		HAD scale
Depression	x		HAD scale
Psychological well-being	x		PWBS
**Sociodemographics**			
Age	x	x	ad hoc questionnaire
Country of origin	x	x	ad hoc questionnaire
Education level	x	x	ad hoc questionnaire
Marital status	x		
Work status	x		ad hoc questionnaire
Profession	x		ad hoc questionnaire
Contract type	x		ad hoc questionnaire
Working hours	x		ad hoc questionnaire
Work shift	x		ad hoc questionnaire
Work location	x		ad hoc questionnaire
Annual income	x		ad hoc questionnaire
**Informal caregiving support**			
Childminder (hours/week)	x		ad hoc questionnaire
Domestic help (hours/week)	x		ad hoc questionnaire
**Early intervention context**			
Type of early intervention centre		x	ad hoc questionnaire
Time in early intervention centre (years/months)		x	ad hoc questionnaire
Attendance at the centre (days/weeks)		x	ad hoc questionnaire
Type of intervention (sessions/week)		x	ad hoc questionnaire
**Health and lifestyles**			
Height	x	x	ad hoc questionnaire
Weight	x	x	ad hoc questionnaire
Medication	x	x	ad hoc questionnaire
Psychological treatment	x		ad hoc questionnaire
Psychiatric treatment	x		ad hoc questionnaire
Medical condition		x	ad hoc questionnaire
Disability		x	ad hoc questionnaire
Sleep (hours/day)	x	x	ad hoc questionnaire
TV watching (hours/day)	x	x	ad hoc questionnaire
Electronic devices (hours/day)	x	x	ad hoc questionnaire
Leisure activities (type, time per day)	x	x	ad hoc questionnaire
Time spent on daily meals (minutes)		x	ad hoc questionnaire
Sedentary activity	x		ad hoc questionnaire
Adherence to Mediterranean diet	x		14-item QMDA
Smoking consumption	x		ad hoc questionnaire
Alcohol consumption	x		ad hoc questionnaire
Physical activity	x		IPAQ-SF

Abbreviations: OBQ-E, Occupational Balance Questionnaire (Spanish version); EMAS, Engagement in Meaningful Activities Survey; HAD scale, Hospital Anxiety and Depression scale; PWBS, Psychological Well-Being Scale; QMDA, Questionnaire of Mediterranean diet adherence; IPAQ-SF, Physical Activity Questionnaire short form.

### Main outcome measures

#### Occupational participation.

Parental occupational participation will be assessed using the Spanish versions of the OBQ-E [37] and EMAS [38].

The OBQ-E is a brief, self-administered, 13-item instrument that evaluates perceived satisfaction with the amount and variety of occupations in which an individual engages. Items can be rated on a 6-point Likert scale ranging from 0 (completely disagree) to 5 (completely agree). The total score, calculated as the sum of item responses, ranges from 0 to 65, with higher scores indicating greater occupational balance.

The EMAS is a self-administered tool that measures the extent to which individuals perceive their daily activities as meaningful. It consists of 12 items, each rated on a 4-point Likert scale from 1 (rarely) to 4 (always). Total scores range from 12 to 48, with higher values reflecting greater engagement in meaningful activities.

### Mental health

Parental mental health will be assessed across four domains: stress, anxiety, depression, and psychological well-being.

The Parental Stress Scale (PSS) [42] is a 12-item self-report questionnaire measuring stress associated with the parenting role. Items can be rated on a 5-point Likert scale (from strongly disagree to strongly agree). It assesses both negative aspects of parenting (e.g., feelings of exhaustion, restriction of freedom) and positive aspects (e.g., emotional rewards, sense of fulfilment). To calculate scoring, some items should be reverse-coded. Higher total scores indicate higher levels of parental stress.

Symptoms of anxiety and depression among parents will be evaluated using the Spanish adaptation of the Hospital Anxiety and Depression (HAD) scale [43]. This is a self-administered instrument designed to assess emotional distress related to anxiety and depression over the past week, a 14-item instrument with two subscales: anxiety and depression (7 items each). Responses are rated on a 4-point Likert scale (0–3). Each subscale yields a maximum score of 21, with higher scores indicating greater symptom severity. Scores of 8–10 suggest borderline symptoms, while scores ≥11 indicate clinically significant distress.

Psychological well-being of parents will be assessed using the 29-item Spanish adapted version of the Psychological Well-Being Scale (PWBS) [44]. This self-administered questionnaire includes six distinct dimensions: self-acceptance (items 1, 7, 19, 31), positive relationships with others (items 2, 8, 14, 26, 32), autonomy (items 3, 4, 9, 15, 21, 27), environmental mastery (items 5, 11, 16, 22, 39), purpose in life (items 6, 12, 17, 18, 23), and personal growth (items 24, 36, 37, 38). Each item is rated on a 6-point Likert scale (1 = strongly disagree to 6 = strongly agree), with negatively worded items reverse-coded. Higher total scores indicate greater psychological well-being.

### Potential explanatory variables

Data on relevant child and parent characteristics were collected through a combination of ad hoc questionnaires and validated instruments, including the 14-item Questionnaire of Mediterranean diet adherence (QMDA) [45] and the International Physical Activity Questionnaire – Short Form (IPAQ-SF) [46], both completed by parents. The selection of these tools, as well as the design of the ad hoc questionnaires, was informed by previous research on sociodemographic and lifestyle factors associated with the main study outcomes [9,15,34,36,47–50]. In addition, the research team relied on their prior experience in previous epidemiological population-based observational studies, such the InProS project [51] and the INMA mother-child cohort [52].

### Qualitative component: participatory research process

The qualitative component of the EQo-Mental project unfolds in two sequential phases: (1) a participatory session with parents, and (2) a multi-stakeholder focus group.

#### Phase 1: Participatory session with parents.

A 150–180-minute participatory session was conducted with parents using structured and creative techniques to explore the loss or reduction of meaningful occupations. The session followed a five-step structure designed to promote reflection and collective action:

- Identification of meaningful activities lost or reduced due to caregiving demands.
- Exploration of the personal significance of these activities.
- Analysis of perceived barriers (personal, environmental, service-level).
- Discussion of potential facilitators and resources.
- Proposal of actions and strategies at individual, community, and policy levels.

Participants worked individually and in small groups using cards, keywords, and visual prompts to support expression and reflection. The session was co-facilitated by two trained moderators and supported by a research assistant.

Data collection methods included audio and video recordings of the session. Moreover, each session was documented using field notes taken by the research assistant, focused on:

- Non-verbal communication and group dynamics.
- Participant engagement and emotional climate.
- Emerging themes or tensions not explicitly verbalised.
- Environmental conditions and logistical aspects.

A post-session quality questionnaire was also administered to evaluate participants’ satisfaction and the perceived usefulness of the session.

#### Phase 2: Multi-stakeholder focus group.

Findings from the participatory session will undergo thematic analysis. Key insights and action proposals will inform a second focus group involving parents, healthcare professionals, and local stakeholders. This phase will serve to validate, enrich, and prioritise proposals and generate a policy brief to be shared with community or regional decision-makers.

### Data management

Data collected from questionnaires will be compiled into a database created using Microsoft Office Excel (Microsoft Corporation, Redmond, WA, USA). To ensure the confidentiality of personal information, each participant will be assigned a distinct identification number. All data will be anonymised and treated with strict confidentiality. A dissociation protocol will be employed to sever the link between identifying personal data (e.g., name) and a randomly assigned code used for analysis. This anonymised code will be the only identifier used by the research team. The key linking participant identity and code will be securely stored in a file accessible only to the principal investigator.

All digital files will be duplicated and stored on a secure, password-protected server dedicated to research use. Original paper materials (e.g., questionnaires, consent forms) will be numerically organised and stored in locked cabinets. Responsibility for data management and secure storage will be delegated to a trained research assistant under the supervision of the principal investigator. All files will be retained for a minimum of ten years following study completion.

For the qualitative component, all audio and video recordings from the participatory sessions and focus groups will be transcribed verbatim and anonymised before analysis. Transcriptions, field notes, and completed quality questionnaires will be securely stored using the same dissociation protocol and coding system as for quantitative data. Digital qualitative materials (e.g., recordings, transcripts) will be encrypted and saved on the project’s dedicated server with restricted access. Paper-based field notes and materials will be organised and stored securely alongside the rest of the project documentation. Only authorised members of the research team will have access to these data.

### Data analysis

#### Quantitative data analysis plan.

Data analysis will be performed using R software (version 4.2.3; R Foundation for Statistical Computing, Vienna, Austria; http://www.r-project.org/). This part of the study addresses aims 1–6, which include the psychometric validation of two occupational instruments and a cross-sectional analysis of occupational and mental health outcomes.

#### Psychometric validation (Aim 1).

The psychometric evaluation of the OBQ-E and EMAS will follow the methodological standards recommended by the COSMIN group [41]. Internal consistency will be assessed using Cronbach’s alpha, including item-level analysis. Values above 0.70 will be considered acceptable.

Test-retest reliability will be evaluated in a subsample (n = 75) over a 15-day interval using Pearson’s or Spearman’s correlation coefficients, depending on variable distribution. Additionally, the Intraclass Correlation Coefficient (ICC) will be calculated. A correlation coefficient > 0.50 and an ICC > 0.75 will be considered indicators of good reliability.

Construct validity will be assessed through hypothesis testing, as recommended by COSMIN. A priori hypotheses include positive correlations between OBQ-E and EMAS scores, and between these scores and psychological well-being, as well as negative correlations with stress, anxiety, and depression. Zero-order correlations will be used to test these hypotheses.

#### Cross-sectional analysis (Aims 2–6).

The normality of continuous variables will be assessed using the Kolmogorov-Smirnov test with the Lilliefors correction. Descriptive statistics for continuous variables will be reported as means with standard deviations (SD) for normally distributed variables, or medians with interquartile ranges (IQR) for non-normally distributed variables. Categorical variables will be summarised as frequencies and percentages.

To compare scores of engagement in meaningful activities, occupational balance, anxiety, depression, stress, and psychological well-being across different sociodemographic groups, statistical tests will be applied: Student’s t-tests (for two independent groups with normally distributed data), Mann-Whitney U tests (for two independent groups with non-normally distributed data), one-way ANOVA (for three or more independent groups with normally distributed data), or Kruskal-Wallis tests (for three or more independent groups with non-normally distributed data).

Multiple linear regression models (classical or robust, depending on model assumptions) will be applied to examine associations between main outcomes (mental health and occupational performance measures) and potential explanatory variables. Model assumptions will be checked (normality, homoscedasticity, multicollinearity), and robust regression will be applied where standard assumptions are violated. Effect estimates will be reported with 95% confidence intervals, and a significance level of p < 0.05 will be adopted.

Potential confounding variables will be identified through a comprehensive literature review. Variables with a p-value < 0.20 in bivariate analyses will be considered for inclusion in the multiple models to adjust for confounding and estimate adjusted effect sizes. Particular attention will be paid to detecting effects greater than 10% in magnitude.

Sensitivity analyses will be conducted to assess the robustness and consistency of the study findings under alternative model specifications or assumptions.

### Qualitative data analysis plan

This part of the study addresses Aim 7, focusing on participatory-action research. The qualitative component of the EQo-Mental project will be analysed using reflexive thematic analysis following the six-phase approach proposed by Braun and Clarke [53], a flexible and rigorous method well-suited to participatory and exploratory research. This approach enables a detailed examination of parents’ experiences regarding occupational loss, imbalance, and their mental health within caregiving contexts.

The analysis will proceed in two stages, corresponding to the two qualitative phases of the project: (1) participatory sessions with parents and (2) a multi-stakeholder focus group. In both stages, data analysis will be supported using ATLAS.ti software (Version 25.0.1; ATLAS.ti Scientific Software Development GmbH, Berlin, Germany; https://atlasti.com/) to facilitate systematic coding, theme development, and data organisation.

#### Stage 1: Participatory session with parents.

1. Familiarisation with the data: The research team will transcribe audio and video recordings, field notes, and quality questionnaires from the participatory sessions. Researchers will immerse themselves in the data, noting initial ideas and analytic impressions.
2. Generating initial codes: An inductive, data-driven coding approach will be employed. Multiple team members will independently code transcripts to identify relevant features related to meaningful activities, occupational imbalance, caregiving challenges, and support systems.
3. Searching for themes: Codes will be collated into potential themes representing patterns in participants’ experiences. This process will include identifying clusters of meaning around barriers, facilitators, emotional responses, and context-specific needs.
4. Reviewing themes: Themes will be reviewed collaboratively to ensure coherence and accuracy in relation to the coded data and the full dataset. This step includes refining and merging themes as needed.
5. Defining and naming themes: Each theme will be clearly defined, named, and supported with illustrative quotations that reflect participants’ voices and lived realities.
6. Producing the report: A thematic map will be constructed, and findings from Stage 1 will be synthesised to inform the next phase. Results will be interpreted in light of the research questions and used to shape the stakeholder focus group.

#### Stage 2: Multi-stakeholder focus group.

Findings from Stage 1 will guide a follow-up focus group involving parents, professionals, and local decision-makers. The group will validate, enrich, and prioritise actions proposed in the participatory session.

1. Familiarisation and coding: Transcripts from the focus group involving parents, professionals, and decision-makers will be reviewed and coded using the refined coding framework from Stage 1. The codebook will be adjusted as needed to incorporate new stakeholder insights.
2. Theme integration and refinement: Themes from both stages will be integrated to produce a comprehensive understanding of needs, barriers, and actionable strategies. Special attention will be paid to areas of convergence and divergence between parents and stakeholders.
3. Interpretation and translation into action: Final themes will be translated into evidence-informed recommendations. These findings will guide the development of a policy brief for local and regional decision-makers.

All qualitative data will be analysed iteratively and reflexively, with close attention to the social, cultural, and emotional contexts shaping parental experiences. This dual-stage participatory analysis will ensure that both parents’ voices and interdisciplinary perspectives are embedded in the final interpretation and knowledge translation process.

### Study timeline

Recruitment for the quantitative phase began in November 2023 and is expected to conclude by October 2025. Data collection has been running concurrently with recruitment, with the goal of completing all quantitative data collection by the same date.

In parallel, participatory processes linked to the qualitative phase started in January 2024. A total of six participatory group sessions have been conducted to date across different centres. Preliminary analyses of these sessions are currently underway. Based on these results, a focus group is expected to be conducted around November 2025.

Quantitative data analysis is ongoing and preliminary results are anticipated in 2026.

## Discussion

This mixed-method study was designed to examine the association between mental health, occupational balance, and engagement in meaningful activities among parents of children with DD, while also identifying their perceived needs and potential strategies to enhance occupational participation. To our knowledge, this is the first time a study applies both an epidemiological and participatory approach to bridge the knowledge gap between parental mental health and occupational participation in the context of caregiving. Unlike previous research, often limited to emotional or psychological impacts, this study offers an integrated framework for comprehensively understanding how caregiving demands affect parental engagement in meaningful occupations. Moreover, we aim for the findings to inform early intervention practices through an innovative model for family-centred research—one that not only supports developmental care of children with DD, but also actively incorporates parents’ voices and addresses their occupational needs.

In Spain, family-centred practices within early intervention, although formally recognised and recommended for more than two decades [54,55], remains inconsistently implemented. Traditional early intervention approaches continue to prioritise the child’s needs, often relegating families to a peripheral role [56–59]. Despite recent efforts to embed the family-centred model into service delivery [60–62], research among professionals highlights persistent weaknesses and challenges in operationalising this paradigm shift [57–59,61,62]. In parallel, research exploring the families’ own perspectives remains limited [58,63–68], and few studies have adopted a participation-based approach to understand the burden of caregiving, the occupational needs of parents, and their potential to play an active role in promoting parent–child interaction within early intervention services [66,68].

In response to this critical research and practice gap, our study places parental occupational participation and mental health at the core of inquiry. Rather than framing meaningful activities and occupational balance merely as outcomes, we conceptualise them as core determinants of well-being, essential for understanding how caregiving demands reshape the everyday lives of parents—particularly through the disruption, substitution, or loss of meaningful occupations. This occupational perspective aligns with the foundational principles of occupational therapy, which emphasise the interplay between occupation, health, and participation across life contexts [69].

A major limitation in current research on parents of children with DD is the lack of mixed-methods approaches that integrate both subjective experiences and population-level insights. Mixed-method designs offer a powerful strategy for addressing complex phenomena in health sciences, particularly in fields like occupational therapy and caregiving, where lived experience, personal meaning, and contextual barriers are central to understanding health disparities and inequities [70]. Their main advantage lies in combining numerical patterns with rich, contextualised narratives, thereby providing a deeper, more holistic understanding of the phenomena under study [70]. This approach is particularly valuable in fields such occupational therapy research and caregiving where lived experience, subjective meaning, and contextual factors are central to understanding the impact of caregiving roles. At the same time, embedding an epidemiological lens within this framework enables the identification of risk factors, measurable patterns, and social determinants of occupational imbalance and mental health—enhancing the generalisability and policy relevance of findings. Building on this methodological approach, the EQo-Mental project presents several additional strengths. First, it combines a robust quantitative component—including a large, diverse sample (n ≈ 700) and the use of validated instruments—with a novel dual-phase qualitative strategy. This includes participatory techniques with parents and a multi-stakeholder focus group, analysed through reflexive thematic analysis to ensure that parents’ voices are not only heard but also translated into co-designed proposals for action. The validation of the OBQ-E and EMAS in this population further strengthens the study’s methodological contribution by providing culturally adapted tools for assessing occupational determinants of health.

Interestingly, this project also contributes to expanding the research scope of occupational therapy into community-centred and population-based practice [71]. This perspective not only supports family-centred care from a rights-based approach, but also reflects the transformative potential of occupational therapy when situated within a public health framework [72]. By mapping occupational needs and promoting co-constructed solutions with families and professionals, EQo-Mental fosters a platform for policy dialogue and participatory action—contributing to the long-overdue integration of parental needs into early intervention services and aligning with international efforts to design health systems “with” and not just “for” the community [71,72].

Nevertheless, this study has several limitations that should be acknowledged. First, the use of self-reported questionnaires for assessing mental health, occupational balance, and engagement in meaningful activities may introduce biases such as social desirability or recall bias. Although we selected validated instruments to mitigate these risks, it is possible that participants’ responses reflect perceived expectations rather than objective experiences. Second, the cross-sectional nature of the main quantitative analyses limits the ability to establish causal relationships between caregiving demands, occupational participation, and mental health outcomes. However, the findings will provide valuable insights into associations and risk factors, offering a foundation for designing future longitudinal studies to confirm directionality and temporal associations. Third, although the project employs a large and diverse sample from early intervention centres in the province of Alicante, the regional focus may restrict the generalisability of the findings to other populations with different socio-cultural or health system contexts. Since early intervention practices are managed at the regional level in Spain, future research should aim to replicate the study in other regions and settings to strengthen external validity. Fourth, while the qualitative component uses a rigorous reflexive thematic analysis, it inherently involves a level of subjectivity in data interpretation. The research team has implemented measures to enhance credibility—such as multiple coders, reflexivity practices, and triangulation—but some degree of interpretative bias remains inevitable in qualitative research. Finally, sustaining stakeholder engagement throughout the participatory process may pose challenges, particularly given time constraints and differing professional and parental priorities. To address this, efforts will be made to maintain open communication, offer flexible participation options, and ensure that stakeholders see clear value in their involvement.

## Conclusions

This study offers an innovative epidemiological and participatory approach to the investigation of occupational balance, engagement in meaningful activities, and mental health in parents of children with DD. Building on available evidence, the EQo-Mental project attempts to address critical gaps in the understanding of how caregiving demands reshape parental occupational participation and impact well-being.

Looking ahead, the findings from this study may serve as a foundation for prospective longitudinal research to confirm causal pathways between caregiving demands and health outcomes. Furthermore, the participatory methods developed for this study could be adapted to explore the occupational needs of other caregiver populations, such as caregivers of adults with disabilities or chronic illnesses, and across diverse cultural and healthcare settings.

Ultimately, by identifying and addressing the occupational needs of parents of children with DD, this project aspires to contribute to the design of future interventions that promote occupational balance and psychological well-being, strengthen family engagement and resilience, and foster healthier developmental environments for children with DD. This approach supports the implementation of more effective and truly family-centred practices within early intervention services.
